# Gluten-Free Products for Celiac Susceptible People

**DOI:** 10.3389/fnut.2018.00116

**Published:** 2018-12-17

**Authors:** Sweta Rai, Amarjeet Kaur, C. S. Chopra

**Affiliations:** ^1^Department of Food Science and Technology, G. B. Pant University of Agriculture and Technology, Pantnagar, India; ^2^Division of Food Science and Technology, Punjab Agricultural University, Ludhiana, India

**Keywords:** anti-oxidant, celiac disease, cereal, gluten, hydrocolloids, wheat

## Abstract

The gluten protein of wheat triggers an immunological reaction in some gluten-sensitive people with HLA-DQ2/8 genotypes, which leads to Celiac disease (CD) with symptomatic damage in the small intestinal villi. Glutenin and gliadin are two major components of gluten that are essentially required for developing a strong protein network for providing desired viscoelasticity of dough. Many non-gluten cereals and starches (rice, corn, sorghum, millets, and potato/pea starch) and various gluten replacers (xanthan and guar gum) have been used for retaining the physical-sensorial properties of gluten-free, cereal-based products. This paper reviews the recent advances in the formulation of cereal-based, gluten-free products by utilizing alternate flours, starches, gums, hydrocolloids, enzymes, novel ingredients, and processing techniques. The pseudo cereals amaranth, quinoa, and buckwheat, are promising in gluten-free diet formulation. Genetically-modified wheat is another promising area of research, where successful attempts have been made to silence the gliadin gene of wheat using RNAi techniques. The requirement of quantity and quality for gluten-free packaged foods is increasing consistently at a faster rate than lactose-free and diabetic-friendly foods. More research needs to be focused on cereal-based, gluten-free beverages to provide additional options for CD sufferers.

Three important species, maize, rice, and wheat, account for about 90% of total cereal production and are the most widely grown and consumed staple foods in the world. In terms of production, wheat is third in order with about 713 million tons grown in 2013, compared to 745 and 1017 million tons rice and maize, respectively (1). Wheat has the widest geographical distribution, being grown and consumed as a staple food in both highly industrialized western countries (Western Europe, North America) and in developing countries (China, Brazil, India). Wheat consumption in food increased from 11.85% in 1961 to 24.41% of total kCal in 2011 in India, and from 12.20 to 17.83% of total kCal in China. The storage proteins of the various cereals have been given common names: gliadins (prolamins) and glutenins (glutelins) of wheat, secalins of rye, hordeins of barley, avenins of oats, zeins of maize, oryzins of rice, and kafirins of millet and sorghum. Out of these storage proteins, the gluten proteins of the various cereals are gliadins (prolamins) and glutenins (glutelins) of wheat, secalins of rye, hordeins of barley, and avenins of oats. According to the Codex Alimentarius, gluten is defined as “a protein fraction from wheat, rye, barley, oats, or their crossbred varieties and derivatives thereof, to which some persons are intolerant and that is insoluble in water and 0.5 mol/L NaCl” (2). The gluten proteins occur solely in the starch rich endosperm of the grains and make up around 70–80% of total grain protein (3). The gluten is fractionated using 40–70% aqueous ethanol for extraction of prolamins which accounts 50% of gluten protein. According to differences in solubility, the gluten proteins had been divided into two fractions, prolamins and glutelins. The prolamin fraction contains mainly monomeric proteins insoluble in water and salt solutions but soluble in aqueous alcohols (e.g., 60% ethanol or 50% propanol). Glutelins are polymerized by interchain disulphide bonds and insoluble in water, salt solutions, and aqueous alcohols. Gluten-free diets should include abstinence from not only wheat but also bread, biscuits, noodles, and other processed foods prepared using rye, barley, and oats. Rice, corn (maize), sorghum, and pearl millet products are safe staples in the diet for such patients. In 2007, the Food and Drug Administration (FDA) proposed norms for labeling gluten-free products and under proposed ruling the term “gluten–free” is voluntary, and a product that contains no gluten needs to state this fact. A product is qualified as gluten-free if gluten content is <20 ppm. For labeling purposes, gluten–free also means the food is free from any ingredients that contain gluten or must have been processed to remove gluten, to a level of 20 ppm or less (4). In the present review an attempt has been made to summarize the issue of gluten intolerance and technological interventions for developing gluten-free products.

## Gluten Intolerance

**Figure 1:**
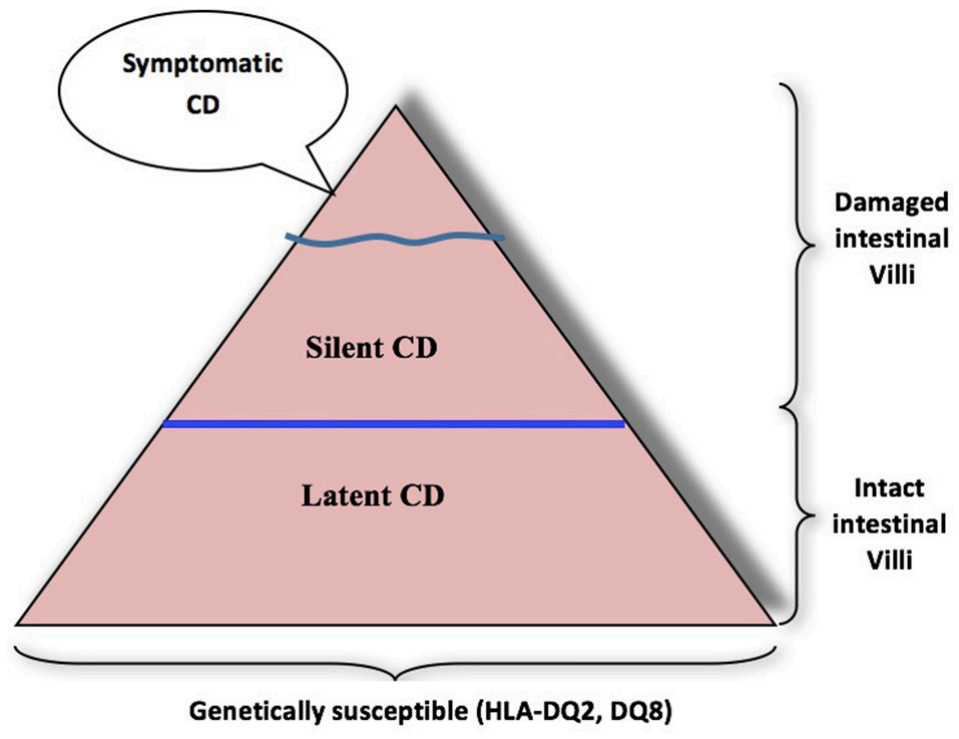
Iceberg for gluten-sensitive people.

Gluten intolerance is an enteropathy triggered by ingestion of prolamine present in wheat, rye, and barley (5). Ingestion of gluten causes serious damage to small intestine mucosa differentiated by inflammation, lymphocytic infiltration, villous flattening, and crypt hyperplasia. Diarrhea, abdominal pain, and weight loss are typical gastrointestinal symptoms of diagnosed active celiac disease (CD); however, the silent form of celiac disease occurs often in adults (6). Celiac disease is significantly associated with certain human leukocyte antigen (HLA) genotypes, as people carrying the DQA1^*^0501 and DQB1^*^0201 (DQ2), or DQA1^*^0301 and DQB1^*^0302 (DQ8) alleles are susceptible (7–9)_._ Gluten proteins are characterized by high glutamine (26–53%) and proline (10–29%) contents, which makes them resistant to human gastrointestinal enzymes (10). The 33-mer peptide from α2-gliadin (amino acid sequence positions 56–88, LQLQPFPQPQLPYPQPQLPYPQPQLPYPQPQPF) contains three overlapping T-cell epitopes (3 × PQPQLPYPQ, 2 × PYPQPQLPY and PFPQPQLPY) for CD sensitive individuals. The human gastrointestinal enzymes pepsin, trypsin, and chymotrypsin were unable to hydrolyze the 33-mer peptide due to their inability to cleave before or after proline or glutamine, leaving the epitopes intact. Comparatively, large CD immunogenic peptides (≥9 amino acid residues) reach the small intestine (11) after crossing through the epithelial barrier and initiate immunogenic cascade in the laminapropria. First, the peptides (≥9 amino acids) are specifically modified by endogenous enzyme tissue transglutaminase (TG2), either by partial deamidation of glutamine residues leading to negatively charged glutamic acid residues or by crosslinking to TG2 by isopeptide bonds between glutamine residues of the peptides and lysine residues of TG2 through transamidation. The subsequent deamidation or transamidation of gluten peptides by TG2 results in increased CD-immunoreactivity compared to unmodified gluten peptides. Deamidation generates gluten peptides with negatively charged amino acid residues that have a higher affinity to HLA-DQ2/8 heterodimers on antigen-presenting cells, which in turn leads to increased CD4^+^ T-cell proliferation. It is well-established that celiac disease is an immune-mediated disorder where intestinal CD4+ T cells are highly reactive to dietary gluten and have a crucial role in disease pathogenesis (12). Recent studies have suggested the pivotal role of both innate and adaptive (CD8^+^ T cells) immune cells in damage to the mucosal tissue of the small intestine (13). Gluten intolerance normally affects young children, but researchers have established that many adults in wheat growing areas are victims of celiac disease. The first accurate clinical description of CD showed that broad flat villi and a dense chronic lymphoepithelial inflammatory cell infiltrate the small intestinal mucosa of patients (14). CD was thought to be a rare disease, with a prevalence of about 0.02%; however, using serology and biopsy, recent studies carried out in Europe, India, South America, Australasia, and USA indicate that the prevalence may be between 0.33 and 1.06% in children and between 0.18 and 1.2% in adults (15). The exclusive treatment for celiac disease is lifelong total avoidance of gluten ingestion by avoiding the consumption of wheat, rye, and barley. There is a growing trend among people who are not sensitive to gluten, but who consciously choose a gluten-free diet in pursuit of a perceived healthier lifestyle. The prevalence of celiac disease can be explained by the iceberg model (16). The overall size of the iceberg is influenced by the frequency of predisposing genotypes in the population and gluten consumption (Figure 1). Accurately diagnosed cases of symptomatic celiac disease are placed on top as the visible section of the iceberg in quantitative terms. This section of the iceberg represents the group consisting the different clinical manifestations of celiac disease. They include both gastrointestinal and extra-intestinal symptoms: the most common are chronic diarrhea, abdominal pain or bloating, vomiting, and weight loss. All patients “above the water” have the characteristic damage of the small intestinal lining (flattening of the villi) with an elevation of their blood antibodies against tissue transglutaminase (“tTG”), and at least one of the genetic markers, HLA-DQ2 or DQ8 known to be necessary in order for celiac disease to exist. A group of “silent” cases of celiac disease are represented “below the waterline,” which have not yet been identified and have flat small intestinal mucosa. These patients show no or very minimal symptoms. These silent cases must take gluten-free diets and are at risk to get moved to the top of the iceberg. At the bottom of the iceberg, there is a group of patients with latent celiac disease who do have the genotype of susceptible genetic markers, HLA-DQ2 or DQ8, but are asymptomatic to celiac disease and are consuming wheat-based food. Serological testing using serum IgA anti-endomysium, anti-TG2 and/or anti-deamidated gliadin peptide antibodies are recommended (5). Despite the benefits of serological testing, it should be mentioned that the prevalence of seronegative CD accounts for up to 10% of all diagnosed cases. For confirmation of serological results, the histological judgment of small intestinal mucosa is commonly regarded as the gold standard for the reliable diagnosis of CD. In the case of doubtful diagnostic results, HLA-DQ genotyping can be used to rule out the existence of CD because of its high negative predictive value.

## Detection of Gluten

Although many methods, such as immunochemistry-based analytical methods, PCR, MS, and HPLC have been utilized for measuring the content of gluten in gluten-free products, only a few are recommended on the basis of sensitivity, selectivity, speed, and precision with easy availability. Currently, R5 antibody-based competitive ELISA is an internationally accepted choice for gluten analysis, whereas monoclonal G12 and A1 are other alternatives for the effective detection of epitopes of gliadin 33-mer prolamins (17). The set limit values for gluten-free food were determined (<20 mg/kg gluten) and the Mendez ELISA R5 method was defined for the determination of gluten (2). Later the Association of European Celiac Societies (AOECS) recommended the R5 Sandwich ELISA (Mendez) for natural and heat-processed foods and the R5 competitive ELISA for hydrolyzed food. The first-generation assay was calibrated to a potentially toxic peptide containing the epitope “QQPFP” of prolamin, although the result, expressed as peptide equivalents, could not be recalculated to prolamin. Now a subsequent second-generation competitive assay is released using a mixture of hydrolyzed prolamins from wheat, rye, and barley as a new calibrator which directly relates to the threshold values of gluten in gluten-free foods given by the Codex Alimentarius Standard (17). But a recent study revealed that the most immunogenic peptides (responsible of 80–95% of immunoreactivity of celiac T cells) reacted to G12, the widely-used R5 antibody-based competitive ELISA, which recognized only around 25% of these immunogenic peptides of a barley beer (18). Similarly, a gluten-free beer (undetectable level of gluten by Competitive ELISA R5) was shown to contain gluten peptides determined by ELISA G12 and by mass spectrometry, which identified immunotoxic peptides for celiac patients (19). The G12 antibody was raised against the hexameric epitope “QPQLPY” of the highly immunotoxic 33-mer peptide of the α- gliadin protein that induces celiac disease (20). This recognition sequence is repeated three times within the gliadin 33-mer peptide. The ELISAs are most commonly used for gluten analysis because of their specificity, sensitivity, and suitability for routine analysis in the absence of an independent reference method. Methods combining mass spectrometry and liquid chromatography (LC-MS/MS) are the most promising non-immunological approaches for accurate quantitation of gluten traces. However, due to its requirement for expensive equipment and expertise, it is not widely used for routine analysis. Reversed-phase high-performance liquid chromatography is used as an independent reference method to determine gliadin, glutenin, and gluten concentrations. The concentration-absorbance curve arrays of flour blends spiked to defined gluten contents revealed that the polyclonal antibody (pAb) ELISA was less affected by the variability of gluten than the R5 and G12 ELISAs. Clear differences in monoclonal antibody (mAb) responses to hexaploid, tetraploid and, especially, diploid wheat species were observed and the pAb ELISA was the only kit to detect gluten from einkorn wheat (21). Recently a stable isotope dilution assay (SIDA) combined with targeted liquid chromatography tandem mass spectrometry (LC-MS/MS) was used for quantitative determination of the 33-mer peptide ranged from 91 to 603 μg/g in flours of 23 hexaploid modern and 15 old common (bread) wheat as well as two spelt cultivars (22). In contrast, the 33-mer was absent (<limit of detection) from tetra- and diploid species (durum wheat, emmer, einkorn), most likely because of the absence of the D-genome, which encodes α2-gliadins. But most of the modern and old wheat flours contained the 33-mer in a range of 200–400 μg/g flour with an overall average of 368 ± 109 μg/g flour. New developments include immunosensors, aptamers, microarrays, and multianalyte profiling for detecting the gluten in food. Recently the potentiometric electronic tongue, which works as a biosensor, has been developed for labeling gluten and may detect 1 mg/kg gliadin from a different medium (23).

## Role of Gluten in Bakery Products

In bread making, gluten acts as a structural protein. The gluten protein can be separated from flour by washing out with running water along with the removal of starch and other minor components. According to the solubility of gluten proteins in alcohol-water solutions, it has been divided into soluble gliadins, providing viscosity and extensibility to dough, and the insoluble glutenins (glutelines) account for the toughness, elasticity, viscosity of dough. The gliadins are monomer and glutenins are polymer with high and low molecular weight subunits. The glutenins fraction of the gluten protein is insoluble in alcohol and exists as polymeric proteins stabilized by inter-chain disulphide bonds. In addition, numerous proteins linked with disulphide bonds are present in gluten either as monomers or as oligomers and polymers, which are enriched with the lowly charged amino acids glutamine and prolamins (24). The high molecular weight (HMW) subunits of glutenin are considered to be the main determinant of the viscoelastic properties of gluten and dough. The contribution of HMW glutenin to gluten elasticity has been associated with its ability to form secondary structure β-type conformations, which have been found to play an important role on the elasticity of gluten. After complete hydration the glutenins become rough and rubbery, whereas gliadin makes a viscous fluid bulk upon hydration. The highly visco-elastic (strong) dough is formed because of the high content of high molecular weight glutenin polymers. The polymeric high molecular weight subunits of glutenin create an elastomeric network for providing a backbone to interact with the remaining subunits of glutenin and with monomeric gliadins (25)_._ The inter-chain disulphide bonds play a key role in stabilizing the network. The properties of gluten become evident after the hydration of flour, which improves the gas holding capacity and creates extensible dough with a high-quality crumb structure for the bread (26). In the absence of gluten, liquid batter is produced, which results in an inferior crumbling texture in the bread, with poor color and qualities after baking. The role of gluten is more important in pasta making, as the gluten creates a tough protein network to prevent the disintegration of pasta during cooking. But the risk of such problems is low during the preparation of gluten-free biscuits and cookies, as the development of a gluten protein network in its dough is minimally required (except semi-sweet biscuits which may require gluten network). The starch gelatinization and super-cooled sugar are mainly responsible for the texture of biscuits rather than a protein/starch network (27).

## Technological Approaches For Miming Gluten in Gluten-Free Bakery Products

The formulation of gluten-free bakery products is still a challenge to both for cereal-cum-baking technologists. Replacing gluten functionality has been a challenge for food technologists. The absence of gluten leads to weak cohesion and elastic doughs which results in a crumbling texture, poor color, and low specific volume in bread. Hence, during the last few years, numerous studies have been attempted for improving the physical properties of gluten-free foods, especially baked and fermented foods, by utilizing the interaction of the many ingredients and additives which could mimic the property of gluten (28). Approaches proposed for obtaining gluten-free baked foods include the utilization of different naturally gluten-free flours (rice, maize, sorghum, soy, buckwheat) and starches (maize, potato, cassava, rice), dairy ingredients (caseinate, skim milk powder, dry milk, whey), gums and hydrocolloids (guar and xanthan gums, alginate, carrageenan, hydroxypropyl methylcellulose, carboxymethyl cellulose), emulsifiers (DATEM, SSL, lecithins), non-gluten proteins from milk, eggs, legumes and pulses, enzymes (cyclodextrin glycosyl tranferases, transglutaminase, proteases, glucose oxidase, laccase), and non-starch polysaccharides (inulin, galactooligosaccharides) (Table 1). Strengthening additives or processing aids has been fundamental for miming gluten's iscoelastic properties (93), where mainly hydrocolloids have been used for building an internal network able to hold the structure of fermented products. Simultaneously with the same intention, different crosslinking enzymes such as glucose oxidase, transglutaminase, and laccase have been used to create a protein network within the flour proteins (94). However, the success of gluten-free products relied on the type of effect of the enzymes as gluten-free processing aids, type of flour, enzyme source, and level. Generally, the combinations of ingredients and the optimization of the breadmaking process have resolved the technological problems, yielding gluten-free products that met the consumer's expectations concerning texture and appearance of the fresh bread (95).

**Table 1:** Research methodology along with references for developing new generation gluten-free products.

**Approach**		**Ingredient**	**References**
Gluten-free flours	Alternate flour	Rice, Corn, Sorghum, Millets	(29–39)
	Pseudocereals	Buckwheat, Amaranth, Quinoa	(32, 40–49)
	Starches	Rice flour and starch, Corn flour and starch, Potato starch, Cassava starch	(31, 32, 38, 41, 46, 50–56)
	Genetically Modified Wheat	Gliadin deleted wheat	(57–60)
	Legume flour	Soy, Chickpea, Pea, cow pea, Others (Carob, Vinal, Beans, Lentil)	(33, 50, 55, 61, 62)
	Other flour	Chestnut, Flaxseed, Chia	(63–66)
Functional ingredients	Hydrocolloids	Agarose, Guar gum, Locust bean gum, Hydroxypropyl-methylcellulose, Pectin, Xanthan gum	(30, 33, 43, 46, 51, 53, 67–71)
	Fiber fortification	Inulin, Rice bran, Pseudo cereals, Psyllium, sugar beet fiber, dietary fiber, Unripe banana flour	(54, 69, 72–78)
Novel processing approaches	Enzymes	Transglutaminase, Protease, Lipase, cyclodextrin glycosyl transferase	(29, 33, 70, 79–83)
	Lactic acid bacteria/Sour dough	Lactic acid bacteria and yeasts	(29, 42, 84–88)
	High pressure/temperature	High pressure and temperature	(63, 89–92)

## Gluten-Free Starches

Gluten-free starches are used as gelling, thickening, adhesion, moisture-retention, stabilizing, film forming, texturizing, and anti-staling ingredients in absence of gluten, where the extent of these properties varies depending on the starch source. In gluten-free products, starch is incorporated into the food formulation to improve baking characteristics such as the specific volume, color, and crumb structure and texture. Corn, rice, buckwheat, waxy high amylose oat, potato, quinoa, sorghum, tapioca, teff wheat, and amaranth have been used as conventional sources of starch, whereas acorn, arracacha, arrowroot, banana, black beans, breadfruit, cana, chestnut, chickpea, cow pea, faba bean, innala, kudzu, lentils, lotus, mung bean, navy bean, oca, pinto bean, sago, taro, tania, white yam, yam, yellow pea have been used as unconventional sources of starch (96). The granule size, surface and composition help in decision-making regarding the method of processing (grinding or extrusion cooking; dehulling, soaking, or germination; autoclaving, puffing, baking, frying, roasting, microwave cooking, or irradiation) for ensuring better hydrolysis and improved gelatinisation behavior of starches, with a lower level of retrogradation of amylose (96).

## The Alternatives of Gluten-Free Cereals/Gluten-Free Flours

Conventionally alternate flours are used for two different reasons: first, to lower or remove the use of wheat for economic reasons in underdeveloped regions or countries and second, to change the nutritional characteristics of a product by protein, vitamins, or mineral enrichment, especially for CD patients. The nutritional quality of bakery products prepared solely using wheat can be improved by adding protein-rich legume flours and other cereal grains. Bread is traditionally produced from wheat flour which is grown globally, but non-wheat growing countries like Gambia, Ghana, Nigeria import wheat or bread to meet their domestic demand. The flours, whole flours, bran products, proteins of legumes, oilseeds, and other minor cereals can be used effectively for nutritional improvement of bakery products. Attempts have been made to enrich bakery products with nutritionally-rich ingredients for their diversification (23, 31, 36, 61, 97). These products also encourage the utilization of non-wheat cereals that are not commonly consumed by many people. Also, the product can be formulated to meet specific dietary requirements, leading to low-calorie bread, high-fiber bread, gluten-free bread, and diabetic bread including protein enrichment. Making bread without any wheat would require a suitable substitute for gluten for CD-susceptible people. Work on gluten-free bread is not new, because dietetic breads for use by patients with celiac disease have been developed using various starches while omitting gluten.

## Gluten-Free Flours

There are many alternate flours with special attributes to replace or minimize the use of wheat in baking. Maize/corn has been used as a preferable replacement of wheat flour for gluten-free food formulation (Table 1). Corn contains a storage protein called zein, which is unrelated to gluten in its primary structure and different than the types of gluten found in the traditional gluten-containing cereals like wheat, barley, and rye. The maize endosperm proteins simply lack the additional elastic high molecular weight glutenin subunits (HMW-GS) function of wheat, and the addition of a minor amount of this or another similar protein would confer viscoelasticity to the mixture. Co-protein, namely HMW-GS or casein (as a non-wheat protein), stabilized the viscoelasticity of the hydrated, heated (to 35°C), and mixed maize zein, as well as held stable the β-sheet content after mixing (98). The β- sheet structures are made from extended β-strand polypeptide chains, with strands linked to their neighbors by hydrogen bond and, due to this extended backbone conformation, β-sheets resisted stretching. Based on these preliminary data it is now hypothesized that the addition of co-protein, such as HMW subunits of glutenin in wheat gluten, improves the viscoelasticity of zein dough systems. The gliadin-zein hypothesis has been supported by a rheological and physicochemical study of the effect of HMW-GS addition to gliadin and zein composites (98), with an attempt made to relate structural and rheological data. This study suggested that the rheological properties of zein improved with the incorporation of high molecular weight glutenine (HMWG) and provided basic information for future investigations on developments for gluten-free products. One study confirmed the improvement of some patients with refractory celiac disease on GFD when a corn-free diet was prescribed (99). The Celiac Sprue Association, the largest non-profit celiac disease support group in the in USA, reported that the zein protein of corn does not cause any allergic reaction in people and corn flour is quite safe as an ingredient in the formulation of gluten-free products such as bread, corn tortillas, chip, and crackers.

Another alternate flour from rice was used for developing hypoallergenic wheat-free foods (Table 1). Rice starches have enormous potential for formulating gluten-free baked products and are commercially available across the globe. As required for special diets, the rice lack gluten, and have low contents of sodium, with high levels of easily-digested carbohydrates. It was reported that bread prepared using white rice flour after incorporating of rice bran improved flavor, but the phytic acid reduced the bioavailability of minerals (100). Different levels of defatted bran and yeast were used in making breads for investigating their effects on the phytate contents, and it was observed that a higher content of bran decreased phytate degradation whereas yeast content had no significant effect. The Phenolic content was highest in violet rice (500.4 mg GAE 100 g) and lowest in white sorghum (52.3 mg GAE 100 g^−1^) flours. However, total anthocyanins were highest in violet, nerone, and black rice flours. FRAP and ORAC antioxidant capacities were correlated to phenolic contents and found to be higher in violet rice flours (101).

Sorghum (*Sorghum bicolor* (L.) Moench) is an essential grain of grass family Graminae and tribe andropoggonae. Sorghum is considered a safe cereal for celiac patients due to its protein being more closely related to maize than to wheat, rye, and barley. The average protein content of sorghum is 11–12%. Sorghum has excellent potential as a functional food ingredient, which was revealed during a comparison of the quality of gluten-free bread of 10 decorticated sorghum flours where significant differences in crumb grain and hardness among the hybrids was observed (67). However, the volume, height, bake loss, and water activity of the breads differed marginally. Increasing xanthan gum levels decreased the volume but increasing water levels increased the loaf-specific volume. In another study the decorticated sorghum flour was explored for gluten-free bread making quality where sorghum flour (70) was mixed with corn starch (30), water (105), salt (1.75), sugar (1), and dried yeast (2), and batter consistency was standardized by varying water levels to set the same force during extrusion. The volume, height, bake loss, and activity of breads differed slightly (67). The protease and amylase activities were measured every 24 h in a Sudanese sorghum cultivar that was germinated for 5 days (102). The functional properties of flours derived from the germinated sorghum seeds were studied and ungerminated seeds were used as a control. Germinated samples had a higher protein solubility, emulsifying activity, and stability compared to the ungerminated control. It was suggested that germination improved the functional properties of sorghum and it would be possible to design new foods using germinated sorghum. In a study of Grains of Butanua, a new Sudanese sorghum cultivar, the grains were germinated for 0, 1, 2, and 3 days and it was observed that contents of starch, protein, oil, foaming stability, bulk density, and least gelation concentration of the sorghum flour decreased, whereas oil absorption capacity, foaming capacity, and emulsion capacity and stability enhanced with an increase in germination time (103). Improved functional properties of sorghum flour by germination of the grains not only make it useful and suitable for various food processing formulations, but also improve the food product quality. The flat bread was made and organoleptic quality was best with a rice: sorghum: black gram (7:7:6) formulation (out of six different formulations) as evidenced from scores awarded by panelists for overall acceptability (104). Therefore, sorghum provided a good alternative for gluten-free bread and food developers have started using sorghum in some food products marketed to consumers who have celiac disease.

Similarly, millets are also being used in food formulations targeted to consumers with celiac disease. Millet refers to a number of different species belonging to the Poaceae family of the order Poales. There are many varieties of millets and the four major types are pearl millet, proso millet, niger millet, and foxtail millet, all of which lack any trace of gluten. Millets are known for their better digestibility without producing any allergenic reaction in consumers unlike wheat. Millets release less glucose over a longer duration of time as compared to wheat and rice. One of the most famous meat-based snack foods in Turkish cuisine, the “kibbeh,” was prepared using millet flour which, maintained nutritional value and sensorial quality as a gluten-free, cereal-based formulation because of its better oxidation stability measured using thiobarbituric acid-reactive substances (37). To date, the use of minor millets in gluten-free product formulation is limited and needs to be explored globally.

## Pseudo Cereals in Gluten-Free Product Preparation

Amaranth, quinoa, and buckwheat are major highly nutritious pseudocereals utilized in the formulation of the gluten-free diet. It was reported that replacing corn starch with amaranthus flour enhanced the protein by 32% and fiber contents by 152% in gluten-free breads without affecting sensory quality (40). Use of both quinoa and amaranth with a dough of increased moisture (up to 65%) significantly improved the bread quality (loaf volume and crumb softness), nutritional values and dietary fiber content (105). Gluten proteins are not present in grains of pseudocereals but albumins and globulin proteins having high biological value are enriched in pseudo cereals. Interestingly, amaranth storage protein has shown complete absence of immune toxicity in celiac patients (106), which has encouraged researchers to improve the structural properties of quinoa and amaranth as an alternate component for the preparation of bread, pasta, and crackers (47, 107). The bread containing amaranth, quinoa, and sweeteners had similar specific volume, firmness and water activity to those of the control bread, but showed higher protein, lipid, and ash contents and a larger alveolar area (47). In an investigation it was reported that bread with 1.9% guar gum (w/w, total flour basis) and 5% buckwheat flour (of all flours and substitutes) mimicked French bread quality attributes (108).

The refined flours or starches that are used in the preparation of gluten-free products are generally poor in quality unless fortified by fiber and other supplements. Gluten exclusion does not create any specific problem but may have low nutritional and biological value. Also, the gluten free dietary foods are reported to have low content of vitamins (vitamins B and D), ions (calcium, iron, zinc, and magnesium), and fiber (109). Furthermore, the risk of developing obesity and metabolic diseases is increased with a gluten-free diet (110). It becomes the responsibility of the dietician to ensure a nutritionally balanced diet for celiac patients consuming gluten-free food. Thus, dietary fiber fortification in gluten-free baked products has been a choice of research for food technologists. Inulin is the most acceptable dietary fiber and acts as a source of non-digestible polysaccharides and prebiotics in gluten-free products. As a prebiotic, inulin stimulates the growth of healthy bacteria in the colon. Further, it was reported that gluten-free bread prepared after the inclusion of inulin (8%) in wheat starch-based formulations increased the dietary fiber content from 1.4% (control) to 7.5% (control + inulin), and the crust color of the bread improved after incorporating inulin (111). The browning of the crust in bread has been attributed to the partial hydrolyzing of inulin by yeast enzymes. As discussed above, the use of pseudocereals and oat in gluten-free formulation increases dietary fiber in products. The dietary fibers of oat are nutritionally enriched with a high content of soluble linked β-D-glucan, which comprise 2–7% of the total kernel weight of oat as the main cell wall component. Other than β-glucan, the oat/oat bran contain a higher sum of total dietary fiber as compared to other gluten-free flours. However, the use of oat in gluten-free products has been an issue of debate as oat contains some fraction of gliadin. The oat prolamins are also rich in glutamine and proline, similar to most of the other cereal proteins. At present, oat represents only ~1.3% of the total world grain production, and its production system is scattered. Oats are generally not gluten-free when produced in a conventional production chain because of regular contamination with wheat, barley or rye. But in the EU (since 2009), the USA (since 2013), and Canada (since 2015) oat products may be sold as gluten-free provided that any gluten contamination level is below 20 ppm (112). Five approved European Food Safety Authority (EFSA) health claims apply to oats, which includes oat specific soluble fibers, the beta-glucans, concern about maintenance and reduction of blood cholesterol, better blood glucose balance with increased fecal bulk and concerns about the high content of unsaturated fatty acids. The seed storage proteins of oat are different from those in wheat, barley, and rye and do not contain immunogenic fragments which induce coeliac disease (CD) in genetically predisposed individuals (113).

Recently, in a large group of CD children, it was reported that prolonged daily intake of a considerable amount of pure oats did not cause any significant change in terms of clinical symptoms, serological parameters, and intestinal permeability (114). Inulin, Chicory flour, and oligosaccharide syrup were used in gluten-free bread formulation, which concluded that 5% inulin increased the volume of bread and decreased hardness and staling rate. The crust was darkened and had the highest quality as revealed from sensory evaluation (115). Further, it has been reported that incorporating 3% (flour/starch weight) Psyllium (*Plantago ovata*) increased overall acceptability of gluten-free breads (116). Incorporation of 5.6% Oat ß-glucan did not change bread volume but the decreased hardness of crumbs and the staling rate (117). An inulin-type fructans (ITFs) mixture (50% inulin + 50% oligofructose) was used and it was concluded that 28% ITFs increased bread volume and the staling rate but decreased crumb firmness, with better sensorial properties than control bread (118).

## Modification of Gluten Peptides For Detoxification of Wheat Gluten

Wheat flours modified to eliminate or reduce the immune toxicity of gluten have been used to prepare pasta and baked products. The large peptides of gluten need to be modified/converted into peptides of <9 amino acid residues to minimize the CD-induced immunoreactivity. This has been achieved through numerous approaches, including exogenous enzyme treatment, use of sour dough/lactic acid bacteria, use of genetically modified wheat, etc.

## Enzyme Treatment

The enzymes obtained from various sources have been used for modifying the immunogenic fraction of gluten protein. The modified gluten network after endopeptidase treatment reduces the technological properties (viscoelasticity) of dough and baked products, which are supplemented by structuring agents such as pre-gelatinized starch, emulsifiers and hydrocolloids. The peptidases other than digestive enzymes exhibited predominantly post-proline and/or post-glutamine cleavage activities, effectively degrading the 33-mer peptide into fragments of <9 amino acids in length. The endopeptidase originating from bacterial origin completely degrades the gluten-immune toxic peptides during the preparation of wheat flour dough (119).

Transglutaminase is another highly functional enzyme obtained from different types of sources such animal tissue, fish, plants, and microorganisms. It was reported that microbial transglutaminase can be used to generate a network-like structure in gluten-free bread (80). The microbial transglutaminase (mTG) types used in baking applications are aminotransferases and are primarily obtained from microorganisms. The mTG (EC 2.3.2.13) from *Streptomyces mobaraensis* is commercially available and used as a texturizing agent in various meat, fish, dairy, legume, soy, and wheat products. In contrast to transglutaminase, the mTG is calcium-independent and favors transamidation over deamidation of peptides, which in turn reduces their binding ability to HLA-DQ2/8. It is achieved by cross-linking lysine ethyl ester through mTG and the inducible inflammatory response of gluten sensitivity is reversed without affecting other aspects of the biological activity of gliadins. Alternatively, the transdamidation of toxic epitopes by tissue-transglutaminase of microbial origin (*Streptomyces mobaraensis*) is done in the presence of lysine methyl ester to detoxify gluten proteins (120). The enzyme cyclodextringlycosyltransferase (CG-Tase) has been used for improving bread structure. Rice bread with good specific volume and very soft crumb texture was obtained by the addition of CG-Tase because of the hydrolysis of starch and a reduction in its retrogradation (29). Further, a comparative study was done using commercial CGTase enzyme and the CGTase produced by *Bacillus firmus* strain 37 for the formulation of gluten-free bread (121). The corn and pinion flours were used for making gluten-free bread and rice flour was used as a control. The addition of the CGTase enzymes of both sources increased the specific volume and improved the texture of the breads. In the sensory analyses by non-celiacs, the best score was given for bread with pinion and rice flours and CGTase from *B. firmus* strain 37, while celiacs awarded the best score to the bread with rice flour only with the commercial CGTase enzyme.

Recently, glucose oxidase (33) and protease enzymes (81) were used in gluten-free bread formulations for improving texture and the sensorial properties of gluten-free bread. It was observed that two different doses of glucose oxidase increased dough consistency and catalyzed the oxidation of glucose to give gluconolactone and H2O2 (33). The H2O2 oxidized sulfhydryl groups present in proteins, inducing protein cross-linking through the formation of disulphide bonds. But α-amylase hydrolyzes α-(1–4) bonds present in starch, producing low molecular weight α-dextrins which finally reduced dough resistance during fermentation. The gluten free bread prepared after treatment with a commercial protease from *Bacillus stearothermophilus* (thermoase) had better quality with good crumb appearance, high volume, and soft texture, depending on the amount of enzymes added (81).

Fungal peptidases from *Aspergillus niger* (EC 3.4.21.26), exhibited post-proline cleavage activity and were highly efficient at degrading CD-active peptides as well as intact α-gliadins, γ-gliadins, and high and low molecular-weight glutenin subunits (122). Cereal peptidases used for gluten degradation were acceptable as food grade, capable of optimizing the hydrolyzing gluten proteins, thermostable and highly active, harvested during the malting procedure and are well-acceptable and beneficial for gluten sensitive individuals (123). Furthermore, peptidases from germinated wheat, rye, and barley grains and bran effectively degraded CD-active epitopes into fragments of <9 amino acids (124). Their activities depended on cereal species, cultivar, germination temperature, and pH value during application (125). The EP-B2, a cysteine endopeptidase (EC 3.4.22) from barley, preferentially cleaves peptide bonds after glutamine, often with proline at the P2 position. The addition of 0.03% of caricain (papaya derived peptidase) to wholemeal wheat bread doughs, followed by bulk fermentation for 5–7 h, resulted in a degradation of about 97% of gliadins, but the traces of the residual gliadin content were still present (126). The grain-associated insects, *Bacillus licheniformis* and *B. subtilis* isolates revealed extracellular peptidase activities and hydrolyzed wheat gluten to a degree of 35–38%. The function of this enzyme was similar to the commercially available endopeptidase preparation Alcalase® (Novozymes, Bagsværd, Denmark) (127). Subtilisin (EC 3.4.21.62) from *B. licheniformis* and thermolysin (EC 3.4.24.27) from *B. thermoproteolyticus* were also effective enzymes for degrading wheat gliadin to non-immunogenic peptides (128). An *Aspergillus niger* prolyl endopeptidase degraded gluten proteins and peptides into harmless fragments (129). Papain (cysteine protease), subtilisin (Protin SD-AY10, serine protease), and bacillolysin (Protin SD-NY10, metallo protease) increased the specific volume of gluten-free rice breads by 19–63% compared to untreated bread (130).

## Use of Sourdough/Lactic Acid Bacteria

The sourdough was prepared by fermenting flour with naturally occurring lactic acid bacteria (LAB) and yeasts. In the mature sourdoughs, the lactic acid bacteria were higher in number (>10^8^cfu/g) than the number of yeasts. Type I sourdough has a final pH of 4.0 at room temperature (20–30°C) and is manufactured by continuous daily refreshments with the aim to maintain the microorganisms in an active state. It takes 2–5 (>30°C) days of fermentation for developing type II sourdough as an acidifier with a pH that is <3.5 after 24 h of fermentation (131). The microorganisms were used in the late stationary phase of growth and exhibited restricted metabolic activity. The type III sourdough, as an acidifier supplement and aroma carrier in bread making, is a dried powder used for fermentation by certain starter cultures. A few reports are available about the use of sourdough for the preparation of gluten-free bread (84, 85).

In one study it was reported that food processing by selected sourdough lactobacilli and fungal proteases may be considered an efficient approach for eliminating gluten toxicity, reducing the gluten level below 12 ppm (119). Further, sourdough fermentation, usually with a mixture of lactic acid bacteria (LAB) and yeasts, is traditionally used to produce leavened bread, especially from rye flour. Lactobacillus sp. are predominant among lactic acid bacteria (LAB) and they produce numerous mixed proteolytic enzymes, including metalloendopeptidases, such as PepO and PepF; aminopeptidases, such as PepN and PepC; dipeptidases, such as PepD; and dipeptidyl and tripeptidylpeptidases, such as the proline-specific Xaa-Pro dipeptidyl-peptidase (PepX) (132). The combination of wheat germination and sourdough fermentation with *Lactobacillus brevis* L62 extensively hydrolyzed wheat prolamin down to <5% of the initial content (133). A cell-free extract of two LABs, *L. plantarum* and *Pediococcus pentosaceus*, had a higher gliadin-degrading capacity (83%) in doughs than the corresponding cell suspension (70%), and complete gliadin degradation without using live LAB may be optimized (134). High molecular weight polymers, namely exopolysaccharides, are produced by lactic acid bacteria in presence of sucrose that mimics physiochemical properties of commercial hydrocolloids or gums, such as the ability to form a network and bind water. It counteracts the negative effects of excessive sourdough acidification and enhances loaf volume, shelf-life, the staling rate, and textural properties of products (129).

## Incorporation of Starches And Gums/Hydrocolloids

The starches obtained from rice, potatoes, and tapioca are gluten-free in nature and used for gluten-free formulations. Since hydrocolloids can mimic the viscoelastic properties of gluten, they are mainly used as an ingredient for gluten-free bread formulations. Xanthan gum and hydroxypropyl-methylcelulose (HPMC) are the most important hydrocolloids for gluten-free bread preparation, as evidenced from research findings. Hydrocolloids and gums contain molecules of long hydrophilic chains and high molecular weight, and have colloidal properties. These are polysaccharides or protein derivatives of fruits, seeds, plant extracts, seaweeds, and microorganisms. Hydrocolloids, or gums, stabilize the product and influence the texture of gluten-free or other products by increasing the moisture content (135). After removing gluten starches, the incorporation of hydrocolloids imparted proper texture and appearance to cereal-based foods in the bakery industry. It was reported that fine white and ground rice flours, in combination with CMC (0.8%) and HPMC (3.3%), yielded good quality gluten-free breads (136). In absence of gluten, the HPMC retains the bread quality through hydration of its dry polymer, followed by swelling and the formation of a gel barrier layer (137). In the case of gums, xanthan and xanthan–guar gum were reported to improve dough structure and finally firmness as well as specific volume in breads (30). The application of chia in baking products acts as a hydrocolloid or substitute for eggs, fat, and gluten and improves the nutritional value (138). Many reports are available about selection and optimization of hydrocolloids for formulating gluten-free products (Table 1).

## Incorporation of Dairy Ingredients

Dairy protein, having low lactose, has long been incorporated into the baking industry for improving nutritional and functional quality along with flavor, texture, and storage time of products. After incorporating dairy-based protein, the handling properties of the batter are enhanced because of increased water absorption. Precaution should still be taken regarding the incorporation of lactose-rich powders during formulation of gluten-free breads for celiacs, because the damaged intestinal villi fail to produce lactase enzyme and, consequently, lactose intolerance could be noticed among those patients. Seven different dairy powders were used for gluten-free bread formulation and reported that high protein- and low lactose-containing dairy powders (sodium caseinate, milk protein isolate) improved the overall shape, volume and crumb texture of breads (139). Whey protein was used for the formulation of gluten-free bread and it was reported that a mesoscopically structured whey protein particle system supplemented after mixing with the starch-mimicked elastic and the strain-hardening properties of gluten (68).

## Genetically Modified (GM) Wheat

The *Triticum monococcum* is a primitive diploid (AA genome) species of wheat cultivated by man and, because of its simple genome, it attracted researchers looking for better nutrition and health in celiac patients. In fact, the AA genome encodes for a lower range of gluten, whereas the modern hexaploid wheat genome (AA, BB, and DD) encodes numerous genes for prolamins. The prolamins originated from *Triticum monococcum* and induced a mild inflammatory effect in celiac patients (140). Genetic engineering is one of the recent alternatives to produce a variety of gluten-free wheat. The bread making wheat *Triticum aestivum L*. contains 50 to 70 different functional genes for the translation of gliadins, which are located on the short arm of chromosomes 1 and 6 and are inherited in blocks. The production of a totally gliadin-free wheat variety is not possible by conventional breeding techniques such as selection or hybridization since the bread-making wheat is allohexaploid, whereas gliadin genes are present on 6 different chromosomes. To overcome this obstacle, seven transgenic lines of wheat were produced using RNAi techniques, and all of them revealed lower levels of γ-gliadins (59). Among these lines the γ-gliadins were reduced by 55–80% in the BW208 lines and by 33–43% in the BW2003 lines. Furthermore, in all three gliadins (α, γ, and ω) protein deleted transgenic lines and a significant reduction in the gliadin content was observed, with an average reduction of 92.2% and a range of 89.7 to 98.1% (59). The RNA silencing/RNA interference is based on the principle of reverse genetics, where the expression of the target gene downregulated in a sequence-specific manner. The peptides derived from α-gliadins are recognized as epitopes by the T-cells of most celiac patients unlike γ-gliadins and glutenins, which are less frequently recognized as epitopes by T-cells (141). Further, it was reported that breads prepared with low-gliadin wheat varieties (E82 and D793) revealed similar breadmaking quality characteristics to normal wheat (60). The sensory analysis revealed a preference for low-gliadin bread over rice bread and statistically comparable levels of texture, flavor, and appearance with traditional wheat flour. Additional genetic diversity was created in the bread wheat through ‘synthetic hexaploid wheat’ (SHW) at various institutes around the world, such as the International Maize and Wheat Improvement Centre (CIMMYT, Mexico), NIAB (UK), and the Commonwealth Scientific and Industrial Research Organization (CSIRO, Australia) (142). The *T. turgidum spp. durum* was hybridized with *Ae. tauschii*, followed by a rescue of the triploid embryo, and subsequently colchicine treatment was applied to double chromosomes for generating hexaploid wheats. Accurate selection of diverse *Ae. tauschii* donors is the key to success that maximizes the D genome variation captured with low CD-toxic gliadins (143). Products of mutation breeding can be released on the market without regulation, where random genome-wide mutations carry favorable mutations in the gene(s) of interest. Ethyl methane sulfonate (EMS) is a chemical mutation approach that results in transitions of G/C to A/T nucleotides. In the context of α-gliadin and γ-gliadin genes, EMS treatment may result in missense mutations within epitopes, which could disrupt binding to the antigen-presenting cells. A set of these hexaploid wheat cv. “Chinese Spring” deletion lines has been used to test the effects of individual deletions on the reduction of CD epitopes and on technological properties. A deletion line missing a 6D α-gliadin locus at the short arm of chromosome 6D (6DS) was found to have strongly decreased mAb responses against Glia-α1 and Glia-α3 epitopes (57). The dough mixing properties and rheology were tested for deleted lines and it was observed that deleting the α-gliadin locus on short arm of chromosome 6 (6DS) resulted in significant loss of technological properties of the dough, but a significant decrease in T-cell stimulatory epitopes was noticed. However, deletion of ω-gliadin, γ-gliadin, and low molecular weight glutenin loci on the short arm of chromosome 1 (1DS) maintained the technological properties of dough along with the removal of T-cell stimulatory epitopes. Becker et al. (144) silenced α-gliadins, eliminating 20 different storage proteins from the grain, whereas Gil-Humanes et al. (59) downregulated gliadins from all groups in bread wheat, with an average reduction of 92.2% in the R5 mAb assay. Therefore, even by reducing the level of gliaden, celiac disease sufferers can enjoy good quality bread. The RNA interference technique had been used for suppressing the DEMETER (DME) gene's homeologs, which are responsible for the transcriptional derepression of gliadins and low-molecular-weight glutenins of bread making wheat “*Brundage 96*.” The results of qRT-PCR revealed a 3.0 to 85.2% suppression of DME transcripts in different transgenic wheat lines (145). The term genome editing refers to novel cutting-edge technologies in which highly specific changes are made to genomes without leaving any foreign DNA. They are based on the use of site-directed nucleases that are engineered to make breaks at specific sequences in the genome. Three major classes of nucleases are being exploited: zinc-finger nucleases (ZFNs), transcription activator-like nucleases (TALENS) and clustered regularly inter-spaced short palindromic repeats (CRISPR) nucleases. The application of genome editing to wheat is still in its infancy, but herbicide resistant canola produced by gene editing has already been launched for growth in North America. Although several research groups are currently exploring the application of genome editing to reduce celiac activity, it is a long-term target because of the challenge posed by the presence of multiple genes and expressed proteins (1).

## Innovative Approaches In the Formulation And Improvement of Gluten-Free Products

Efforts for preparing gluten-free products have long been initiated, and marketing of those products has recently gained momentum. Several attempts have been made to develop acceptable gluten-free products by using various types of raw materials like corn flour and starch, rice flour, buckwheat flour, sorghum, millet tubers like potato, and cassava. It is well-established that gluten-free diet products are poor sources of minerals (such as iron), vitamins (such as folate, thiamine niacin and riboflavin), and fiber; therefore the nutritional content of gluten-free foods is an increasing area of concern. In fact, common nutrient deficiencies in coeliac subjects at diagnosis are calorie/protein, fiber, iron, calcium, magnesium, vitamin D, zinc, folate, niacin, vitamin B12, and riboflavin (146). It has been reported that the adding gluten-free alternative grains, including oats and quinoa, positively impacts the nutrient profile (fiber, thiamine, riboflavin, niacin, folate, and iron) of gluten-free diets (147). Different iron compounds were used to fortify amaranth-based gluten-free bread and it was observed that ferric pyrophosphate with emulsifiers, followed by ferric pyrophosphate alone, yielded the most acceptable products (148). Supplementation of 2% and 1.3% calcium citrate and 0.7% calcium caseinate significantly increased the calcium levels in gluten-free bread, which then scored higher regarding overall preference as compared to the control breads (41). Further, it was revealed that commercial breads with formulae high in starch had a high staling rate due to rapid onset of starch retrogradation in comparison to wheat breads or dairy ingredients-based breads with high-fiber ingredients. The lower staling rate of wheat bread in comparison to gluten-free breads may be ascribed to the creation of an extensible protein network developed by gluten followed by a slow retardation of free water resulting in a softer crumb and firmer crust. The xanthan gum and xanthan plus konjac gum were hydrocolloids used for developing two gluten-free bread recipes based on brown rice, soy, buckwheat flour, skim milk powder, whole egg, potato, and corn starch. All the gluten-free breads were brittle after 2 days of storage; they were fractured as cohesiveness, resilience, and springiness decreased significantly (*P* < 0.01) as was revealed from a texture profile analysis, although the keeping quality of the breads improved (86). The proper sequence that developed the proper dough characteristics was dependent on the blending and baking equipment and processing techniques. Gluten-free dough was more fragile and more susceptible to overworking. Chemical leavening and proofing conditions were directly linked to formulation. Oven temperatures generally needed to be lower, while baking times were longer. The ability to control atmospheric pressure might positively influence the performance of gluten-free dough during baking. In an investigation, three different gum types (guar gum, xanthan gum, locust bean gum) were added at a level of 1%, and flaxseed was incorporated at different concentrations (0, 2.5, and 5%) to the formulation. After frozen storage at −20°C for 10 days, the samples were thawed at +4°C. Thawed samples were then fermented and baked in an infrared-microwave (IR-MW) oven. The quality of gluten-free breads formulated with guar gum-5% flaxseed was found to be better (with lower hardness, higher specific volume, and better color) than the other samples, with the lowest hardness occurring during the 72-h storage (63). The extrusion-cooking process is one of the most suitable technologies for gluten-free pasta-making, where native flour is treated with steam and extruded at more than 100°C for a short time in order to promote starch gelatinization directly inside the extruder-cooker. The first technological approach used for production of gluten-free pasta is focused on the use of heat-treated flours, where starch is gelatinized (149). Further, annealing has often been applied to starch as a physical treatment to change its natural physicochemical properties in order to meet different industrial requirements during gluten-free food formulation (149). Specifically, the annealing consists of treating starch with more than 40% water at a lower temperature (50–60°C) of gelatinization and, consequently, a heat-moisture treatment (treatment at small moisture and great temperatures, 100–120°C for rice) improved starch crystallinity, granule rigidity, and polymer chain associations (150). These specific hydrothermal treatments inhibit granule swelling, retard gelatinization and increase starch paste stability, leading to enhanced texture properties and cooking behavior in rice noodles (151). In one study, roasting quinoa seeds at 177°C for 15, 30, and 45 min, improved final viscosity/paste stability and setback/degree of retrogradation after heating, shearing, and cooling, which yielded the best sensory scores for appearance, color, and texture (109, 152) Physical treatments of wheat using microwave (89) or pulsed light (153) irradiation have been recently proposed to reduce the immunoreactivity of gluten proteins. In these cases, the reduction of gluten immunoreactivity has typically been assessed by sandwich ELISA (e.g., R5-antibody ELISA). However, contradicting the preliminary claims that microwave-based treatments of wheat kernels detoxify gluten, it was reported that microwave-based treatments neither destroy gluten nor modify chemically the toxic epitopes (120). This study provides evidence that beside R5-antibody ELISA, other methods like G12 antibody-based ELISA, *in vitro* assays with T cells from gut mucosa of celiac subjects, and Raman spectroscopy must be used to determine gluten level in thermally treated wheat products. Recently, gluten-free breads were prepared after replacing 10% of the starch by the ingredients albumin, collagen, pea, lupine, and soy protein and revealed that bread with pea protein was the most acceptable among different analyzed samples, while breads based on soy protein had the lowest level of sensory acceptance (61). Pea proteins significantly affected the rheological properties of thedough and structure of the bread. Use of structure-forming agents such as hydrocolloids, including guar gum and pectin, requires additional testing in starch-based gluten-free bread formulation (61). Recently, the water extract of linseed has been used as a structure-forming agent in gluten-free baking for assessing their influence on the rheological properties of the dough and quality of the bread, especially its staling rate (154). The replacement of guar gum and pectin with linseed mucilage improved sensory acceptance of the bread and had limited influence on the texture and staling of the bread. The orange pomace in gluten-free bread baking was optimized at a level of 5.5% and a further increase in its content decreased the bread's specific volume (155). The effect of adding teff flour (5, 10, and 20%) and different dried (buckwheat or rice) or fresh (with *Lactobacillus helveticus*) sourdoughs on the sensory quality and consumer preference of gluten-free breads was investigated. The combination of teff (10%) with cereal sourdough (rice or buckwheat) enhanced bread aroma, increasing the fruity, cereal, and toasty notes. High levels of teff (20%) and *Lb. helveticus* sourdough induced a decrease of the loaf area. The visual appearance of breads with 20% teff was most acceptable, while bread combining 10% teff and rice sourdough was preferred in terms of flavor by consumers (156). Because CD is associated with a high incidence of type I (insulin- dependent) diabetes mellitus, the maintenance of a good glycemic control for gluten-free diet is an important task for individuals simultaneously suffering/susceptible with CD and insulin-dependent diabetes. It was demonstrated that 8%. Enriching with inulin type fructans (ITFs) decreased the glycemic response of gluten-free bread, resulting in a low-glycemic index product that combined high acceptability and a physiologically significant supply of prebiotic-soluble dietary fibers (118). Effects of the rice flour particle size and dough hydration level were assessed on the physical properties and the predicted glycemic index (pGI) of gluten-free bread, where the pGI ranged from 61 to 65 (medium GI). The added water content affected the glycemic index, but particle size did not affect the pGI (34). The effects of the different germination times of brown rice flour were investigated as they related to the nutritional quality of brown rice flour-based gluten-free bread (157), and concluded that *in vitro* starch digestibility assay, soaking (pre-germination), and germination can be reduced the hydrolysis index and pGI of gluten-free bread. The unripe banana flour was added (30%) to a blend of rice flour and wheat starch to improve the resistance starch content of their gluten-free bread (72) and recommended 15–65% unripe banana flour, 55–75% buckwheat flour, up to 96% sorghum flour, and up to 100% chickpea flour (fwb) to be formulated into a gluten-free bread with good physical properties and sensory acceptance. In one study, the combination of chestnut flour (40%) and sourdough (20%) fermentation on chemical, technological, and nutritional attributes of gluten-free breads was evaluated, and it observed that chestnut flour limited the acidification of both dough and breads. The volume of all breads prepared with chestnut flour and/or sourdough was lower compared to the control, but the combination of chestnut flour and sourdough contributed to a reduction in crumb grain heterogeneity. Sourdough and/or chestnut flour addition caused a significant increase in crumb hardness probably due to the lower volume. It was reported to produce a gluten-free bread enriched with a significant amount of teff (25%), improving the nutritional properties of the control gluten-free bread (64). Fermentation of teff flour significantly increased the nutritionally relevant soluble fiber and decreased free sugars. The bread enriched with fermented teff had improved the physical properties and led to a lower staling rate as compared to a non-enriched control or non-fermented teff enriched bread. The addition of soy protein isolate (1, 2, and 3%) or egg white solids (5 and 10%) to the HPMC-treated rice cassava bread reduced dough stability by suppressing HPMC functionality, altering water distribution within the dough, weakening HPMC interactions with the starch matrix and reducing foam stability (158). However, when egg white solids were included at a level of 15%, it overcame negative interactions with HPMC and improved loaf volume and crumb regularity by forming an inter-connected honeycomb matrix.

Response surface methodology (RSM) is a statistical tool which has been used by researchers for developing gluten-free products by optimizing the level of ingredients. In statistics, RSM explores the relationships between several stimulus/explanatory variables and one or more response variable (quality parameter). The RSM has been applied to investigate the interacting effects of different levels of ingredients and water on gluten-free dough and bread properties. Further, along with the level of ingredients, pressure, and heat treatment is also optimized in RSM. This approach allows optimizing formulas based on statistical modeling. The RSM was used to optimize the formulation of non-gluten pasta prepared using modified starch, xanthan gum, and locust bean gum and reported similar characteristics of gluten-free pasta to wheat-based pasta (159). It provided a good “hardness of first bite” and cohesiveness to gluten-free pasta. The RSM optimized the proportions of corn starch, cassava starch, and rice flour (corn starch 74.2%, rice flour 17.2%, and cassava starch 8.6%) in the production of gluten-free breads and reported that the addition of soy flour improved the bread crumb characteristics (160). After optimizing a 2.2% HPMC and 79% water flour/starch base (fsb) through RSM, it was observed that crumb and crust firmness increased while the crumb moisture content decreased in gluten-free bread (161). Using response surface methodology (RSM), two different sorghum hybrids and three different protein sources, i.e., soy flour, skim milk, and egg powder, were used to formulate gluten-free breads after incorporating different levels of enzymes (0, 0.01, 1, and 10 U of transglutaminase per gram of protein) for better loaf volume, crumb characteristics, and overall quality as well as the creation of a stable protein network (80). Recently, the RSM was used to optimize a rice flour-based formulation for making gluten-free bread, consisting of 15% carob flour, 15% resistance starch, 10% protein, and 140% water (fwb) (62). In one study, RSM was used to define the optimum HPMC, yeast b-glucan, and whey protein isolate levels in a rice-based gluten-free bread formulation, considering comparable physical properties in wheat bread. The optimal formulation contained 4.35% HPMC, 1% b-glucan, and 0.37% whey protein (162).

The addition of bee pollen (1, 2, 3, 4, and 5%) appears not to have any influence on the rheological characteristics of the enriched doughs when compared to the control. The technological features such as volume, textural properties of crumb, crust and crumb color, crumb cell uniformity, and crumb grain structure significantly improved by increasing the levels of pollen supplementation in gluten-free breads. The fortified breads were softer and showed a slower firming kinetic than the control bread. The gluten-free breads fortified with bee pollen between 3 and 5% had higher overall acceptability (163). Recent (2011 onwards) and important formulations of gluten-free products are enumerated in Table 2, along with applied principles and salient findings.

**Table 2:** List of recent important formulations of cereal based gluten-free products (2011 onwards).

**SN**	**GF product**	**Approach**	**Salient findings**	**References**
1	Bread	Used Rice as alternate flour with pseudo cereal and Hydrocolloids	Blend of rice-buckwheat in proportion of 60:40 was used by incorporating Xanthan gum and propylene glycol alginate at levels of 0.5 to 1.5%. The incorporation of both hydrocolloids significantly improved the storage modulus. As compared to Xanthan gum the inclusion of propylene glycol alginate increased specific volume but decreased crumb firmness and crumb structure of gluten-free breads.	(43)
2	Bread	Red sorghum flour was supplemented with different starch	Gluten-free sorghum bread was prepared using cassava, corn, potato, or rice starch and sorghum in the different ratios. Water (100%), sugar (6.7%), egg white powder (6%), fat (2%), salt (1.7%), and yeast (1.5%) were other baking ingredients. Increasing starch content decreased crumb firmness and chewiness but cohesiveness, springiness, and resilience increased in all breads. The cassava-sorghum and rice-sorghum breads had better crumb properties than corn-sorghum or potato-sorghum breads.	(31)
3	Bread	Rice flour was supplemented with acetic acid, lactic acid, citric acid	In order to improve the quality of gluten-free bread, several levels of acidic food additives (acetic acid, lactic acid, citric acid, and monosodium phosphate) was tested for gluten-free breads prepared using rice flour and hydroxypropylmethylcellulose (HPMC). It was found that monosodium phosphate yields bread producing better texture scores and yielded the highest volumes of the loaf.	(84)
4	Bread	Non-yeasted gluten-free dough mixtures were prepared by mixing wheat starch with locust bean gum-supplemented with whey protein	Reported about robustness of mesoscopically structured whey protein particle system contributing elasticity and strain hardening properties after mixing with starch up to a certain level. Although whey particles provided lower extensibility but were more stable than gluten particles. For reducing the stiffness of dough for better bread making, the N-ethylmaleimide (NEM) treatment blocked formation of excessive number disulphide bonds (essential for bread making dough) of a mesoscopic whey protein.	(68)
5	Bread	Corn starch, potato starch, pectin, sunflower oil, fresh yeast were the basic ingredients of gluten-free bread. Calcium fortification was done	Palatability of gluten-free breads fortified with calcium was significantly higher than those of unfortified control. The addition of calcium caseinate and calcium citrate improved desirable sensory attributes (sweet odor, butter bread odor, butter bread taste, and springiness) for the consumers in comparison to unfortified control. The crumbs of calcium-fortified breads were softer and more elastic.	(41)
6	Bread	Rice flour supplemented with different grades of pseudo cereals	Dough made with higher amount of light buckwheat flour (LBF) or wholegrain flour (WBF) with rice flour resulted in final products with higher antioxidant properties. Bread prepared using whole grain buckwheat flour had higher antioxidative activity than bread prepared with light buckwheat flour.	(44)
7	Bread	Rice flour was supplemented with egg protein, fat, dietary fiber (DF)	Rice bran containing gluten-free breads improved protein and dietary fiber content and the ratio of insoluble dietary fiber to soluble dietary fiber. A rice bran source with high soluble dietary fiber content increased sensory acceptance and shelf life.	(77)
8	Bread	Range of commercial gluten-free flours was used	Breads produced only from oat flour were of similar quality to wheat bread, and the utilization of buckwheat, rice, maize, quinoa, sorghum and teff flours resulted in breads of inferior quality.	(32)
9	Bread	Sorghum flour with sourdough was used and exopolysaccharides incorporation as hydrocolloids	The sucrose formed Exopolysaccharides (EPS) during sourdough fermentation, improving the technological properties of gluten-free breads and had the potential to mimic hydrocolloids. The EPS yielded softer crumbs in the fresh and stored sorghum bread. Among EPS, dextran revealed the best shelf life improvements. All strains (Dextran forming *Weissellacibaria* and reuteran/ fructan forming *Lactobacillus reuteri*) produced oligosaccharides during sorghum sourdough fermentation, contributing to the nutritional benefits of gluten-free sorghum bread.	(85)
10	Bread	Used emulsifiers (diacetyl tartaric acid ester of monoglycerides and sodium stearoyllactylate), hydrocolloids and enzymes (glucose oxidase and α-amylase) in gluten-free bread formulations	The electrophoretic pattern of dough extracted proteins changed with glucose oxidase addition. Contrary to widespread opinion, it was observed that the presence of additives is not essential for gluten-free bread production. It suggested to find new alternate raw materials and technological parameters for facilitating gluten-free product development at lower cost.	(33)
11	Bread	Dough prepared using corn starch, potato starch, guar gum, pectin, and freeze-dried yeasts. Native starch was replaced	The native starch was replaced with high amylose corn starch (HACS), acetylated distarchadipate (ADA), and hydroxypropyldistarch phosphate (HDP). Incorporation of 10 or 15% of HDP or ADA increased volume of gluten-free loaves. On the day of baking a slight decrease in hardness and chewiness of the crumb was noticed whereas HACS deteriorated structural and mechanical properties of the crumb.	(51)
12	Bread	Corn starch was used in combination with various cereal-based proteins	The chickpea-based bread exhibited had better physico-chemical characteristics and sensory behavior than soya protein with other legume protein-based gluten-free breads.	(50)
13	Bread	Corn flour with tapioca starch was used and supplemented with plant and animal protein	A blend of tapioca starch and corn flour (80:20) was used with bread ingredients water, yeast, salt, and sugar. The optimum bread had highest levels of fat and soybean flour and one egg, presented low values of firmness (≤100 N) and elasticity (>65%) and the lowest variation of these parameters with storage. Therefore, tapioca starch-based breads had spongy crumb, high volume, and a good sensory acceptance.	(164)
14	Bread	Used corn starch as alternate flour followed byincorporation of proteins.Response surface methodology	The gluten-free bread prepared by using ingredients albumin, collagen, pea, lupine and soy protein to replace the starch in a gluten-free formulation and revealed that bread with pea protein was most acceptable among different analyzed samples, while least sensory acceptance was observed in the case of a product with soy protein	(55, 61)
15	Bread	Rice flour was used along with different combinations of alternate flours supplemented with hydrocolloids	The HPMC had linearly a positive effect on volume of teff and corn breads and a negative linear effect on this parameter in rice breads, without change in volume of buckwheat bread. Xanthan addition had most linearly negative effect on loaf volume of all breads. HPMC addition reduced crumb hardness of teff, buckwheat, corn, and rice bread, whereas Xanthan increased the crumb hardness of teff and buckwheat breads, but rice breadcrumb remained uninfluenced.	(165)
16	Bread	Used millet and pseudocereals	The *in vitro* starch digestibility of five gluten-free breads (from buckwheat, oat, quinoa, sorghum, or teff flour) was analyzed using a multi-enzyme dialysis system. Quinoa bread showed highest predicted glycemic index (GI 95) followed by buckwheat, teff, sorghum, and oat (GI 71) breads. Larger granule diameters in oat and sorghum in comparison to quinoa and buckwheat flour resulted in lower specific surface area of starch granules and smaller starch granules result in a higher glycemic index.	(35)
17	Bread	Corn starch, rice flour, rice starch were supplemented with psyllium and sugar beet fiber	The syllium, sugar beet fiber improved doughs, but psyllium had a pivotal role on gluten-free bread preparation because of its film-forming ability and had more effective anti-staling effect with water binding capacity.	(54)
18	Bread	Used rice flour treated with protease	Bread prepared from rice treated with a commercial protease from Bacillus stearothermophilus (thermoase) was of higher quality, i.e., good crumb appearance, high volume, and soft texture, depending on the amount of enzyme added. Many cellular structures were formed in the thermoase-treated bread which was missing in the untreated control. Bread crumb color was not affected by the treatment and staling rate was much lower for the thermoase-treated bread than for the control.	(81)
19	Bread	Used rice as alternate flour	The coarse fraction after higher dough hydration (90–110%) improved volume and crumb texture was of rice bread. Slowly digestible starch (SDS) and resistant starch (RS) increased in the coarse flour breads.	(34)
20	Bread	Different types of rice flour were used	The finest rice flours led to poorest retention of the gas produced during fermentation and produced breads with a lower specific volume in both formulations. Along with larger particles a consistent film was formed of water, hydrocolloid, and starch granules fragmented during milling and kneading as revealed from analysis of dough microstructure.	(166)
21	Bread	commercial GF bread mixtures (coded GF1 and GF2) partially replaced with pseudocereals with supplementation of hydrocolloids	For improving the nutritional value of the final gluten-free breads without decreasing their technological quality a dehulled (DBF) and a puffed (PBF) buckwheat flour were used. Inclusions of 40% DBF improved the baking performances of the commercial gluten-free mixtures. Small amount of PBF, as well as of HPMC, resulted in softer gluten-free bread crumbs by reducing both the diffusion and the loss of water from the bread crumb and the interactions between starch and protein macromolecules, and finally reduced staling kinetics during storage.	(46)
22	Bread	Rice flour and potato starch mixtures (1:1) with oat and quinoa malts were used	The incorporation of oat malt decreased batter viscosities of dough and density of bread with formation of more open crumb, but overdosing of oat malt resulted in excessive amylolysis during baking and deteriorated the product. Quinoa malt had no significant effect on the baking properties due to low α-amylase activity.	(52)
23	Flat bread	Use of alternate flour supplemented with Xanthan gum	Rice flour:corn starch:potato starch in ratio of 40%:20%:40% followed by rice flour:corn starch:potato starch in ratio of 40%:40%:20% with 3% xanthan were the best formulations for production of gluten-free Egyptian balady flat bread.	(53)
24	Bread	Corn starch and potato starch were used as main ingredients supplemented with inulin	Inulins with different degree of polymerization were used for the production of gluten-free bread and it was found that addition of inulin increased loaf volume and reduced crumb hardness. Inulin preparations with lower degree of polymerization had better effect on all analyzed parameters.	(73)
25	Bread	Used unripe banana flour and potato starch with rice flour	Evaluated the feasibility of gluten-free bread making using unripe banana flour, a source of resistant starch and dietary fiber, as a starch-based ingredient. Promising results have been found for breads produced with 100% unripe banana flour and combinations involving rice flour and potato starch.	(74)
26	Bread	Used rice flour treated with protease	It was found that rice bread swelling was remarkably improved with a longer period pre-fermenting using *Aspergillus oryzae* at 55°C. After 12 h fermentation, without polymeric thickeners the specific loaf volume was ~2.2-fold higher than that after 0 h. An enzymatic assay of the batter indicated that protease activity during the pre-fermentation period increased which correlated with bread swelling. Furthermore, a commercial protease obtained from *A. oryzae* had an increased batter viscosity and decreased flour settling behavior.	(82)
27	Cookies	Used alternate flours	Investigated rice flour, corn meal, sorghum flour, pearl millet flour, and ground nut meal substitutions in wheat flour, from 0 to 100% each, for the production of gluten-free cookies. Sensory evaluation revealed that the best levels of incorporation for cookie making were rice flour 50 percent, maize meal 50 percent, sorghum flour 50 percent, pearl millet 50 percent, and groundnut meal 20 percent.	(36)
28	Muffins	Used plant protein	The incorporation of different protein isolates (kidney bean, field pea, and amaranth protein) enhanced batter viscoelasticity and resulted in muffins with higher specific volume, springiness, and cohesiveness as compared to wheat gluten. Kidney bean protein isolates resulted in firmer muffins compared to those made from field pea, amaranth, and wheat gluten protein.	(97)
29	Bread	Used genetically modified wheat deleted for gliadins genes	Breads prepared with flour from low-gliadin wheat varieties (E82 and D793) showed breadmaking quality characteristics similar to those of normal wheat flour.	(60)
30	Cookies	Used rice as alternate flour followed by incorporation of fibers	Different fibers (by replacing rice flour up to 20%), added individually or in combination, to improve the functional properties of gluten-free layer cakes. It was observed that gluten-free cakes containing blends of oat fiber-inulin resulted in improved specific volume and other desirable cake making properties.	(167)
31	Muffins	Used rice as alternate flour and incorporated proteins (Soy protein isolate, pea protein isolate, egg white protein, and casein)	Compared the rice muffins produced by using five different protein sources: soy protein isolate, pea protein isolate, egg white protein, casein, and for comparing purposes vital wheat gluten (VWG). The egg white protein increased the height and specific volume whereas color of muffins was dominated by the color of the added proteins. The pea protein isolate containing muffins was the softest and springier than the No-Protein and casein gave the hardest muffin. In general, muffins with best visual appearance were those containing egg white protein or casein.	(168)
32	Biscuits	Used alternate flours	The gluten-free biscuits were prepared using maize flour (MF), rice flour (RF), and soybean flour (SF) after supplementing other ingredients such as palm tree oil, honey, maize starch, eggs, sugar powdered, vanilla essence, and sodium bicarbonate. The blend with flour levels 30:30:40 (MF:RF:SF) led to the highest acceptability.	(169)
33	Pasta	Used sorghum, rice, corn flours with incorporation of potato starch	Fifteen formulations (Sorghum/rice/potato) subjected for sensory analysis and seven formulations were selected in respect to taste and grittiness. Formulations IV (Sorghum/rice/potato/5:2.5:2.5), V (Sorghum/rice/potato/4:2:4), and VI (Sorghum/rice/potato/4:3:3) showed the best sensory results and were tested to chemical analysis and cooking evaluation. The spaghetti-type pasta, obtained in this study, that contained sorghum, rice, and potato flours (proportion of 40:20:40), was the sample that showed the best results in the cooking quality tests. This formulation showed the best density, yield, weight increase, and the lowest loss of solids.	(38)
34	Cookies	Use of yellow corn, buckwheat and rice as alternate flour	Coarse-grained rice flour-based cookies had larger diameter, spread factor, darker color, and lower hardness but rest of the gluten-free cookies (yellow maize, buckwheat) had a lower spread ratio and greater hardness than wheat cookies.	(170)
35	Breads	Used germinated brown rice	After 24 h of germination the brown rice flour-based bread had improved texture which might be ascribed to the increase of amylase activities as well as starch degradation resulting in hydration and pasting. However, excessive germination (beyond 24 h of germination) deteriorated the product as a result of extensive amylolysis.	(157)
36	Cookies	Used pseudocereals of different stages of germination	Cookies from raw and germinated amaranth grain flour were prepared and it was reported that the use of germinated grain decreased fat but increased protein and fiber content. Pasting properties decreased but functional and antioxidant properties improved with germination. Raw amaranth flour cookies showed the highest spread ratio, followed by cookies prepared from germinated amaranth flour and wheat flour.	(48)
37	Bread	Used pseudocereals followed by incorporation of sweetener	Bread prepared using amaranth, quinoa, and sweeteners (sucralose and sucralose-acesulfame) had almost similar specific volume, firmness and water activity to the control bread, but showed higher protein, lipid, and ash contents and a larger alveolar area.	(47)
38	Muffins	Alternate flour incorporated with cow pea protein	Incorporation of white cow pea protein isolates (4–12 g/100 gm rice flour) decreased flour paste viscosities (peak, breakdown, and final) and increased batter viscoelasticity (above 8 g/100 g incorporation level). At above 8 g/100 g incorporation levels firmness, springiness, cohesiveness, and chewiness of the muffins increased for both white and red cow pea protein isolates. The white cow pea protein isolates increased muffin volume whereas red cow pea protein isolate decreased it.	(49)
39	Muffins	Used rice flour blended with varying levels of jambolan fruit pulp (JFP) and xanthan gum (XG)	Blend of jambolan fruit pulp (JFP) and xanthan gum (XG) were used in preparation of rice flour-based gluten-free eggless muffins and it was observed that the incorporation of JFP and XG increased batter viscoelasticity. JFP incorporation increased greenness, cohesiveness, resilience, water activity (aw), total phenolic content, total flavonoid content, DPPH, and ABTS inhibition of the muffins. Further, XG improved muffin quality characteristics (appearance, specific volume, and resilience).	(69)
40	Pasta	Used rice as alternate flour followed by incorporation of bean protein and fiber	Gluten-free spaghetti was prepared using rice flour and different concentrations of white-seeded low phytic acid and lectin-free bean flour (included at levels of 0, 20, and 40%, w/w). The gluten-free rice spaghetti reduced the total starch and the *in vitro* glycemic index, while it increased protein, ash, dietary fiber, and resistant starch contents. It increased the optimal cooking time as well as the water absorption capacity without compromising with cooking loss and texture properties as compared to control spaghetti.	(75)
41	Bread and cake	Used sorghum as alternate flour followed by heat treatment	Heat treatment to sorghum flour at two different temperatures (95 and 125°C) for 15, 30, and 45 minutes was applied and it was observed that heat-treated cake and bread had better acceptability than the controls. Heating the flour at 125°C for 30 min produced bread with the highest specific volume (3.08 mL/g) and the most cells per slice area (50.38 cells/cm^2^). Cakes produced through this treatment had highest volume (72.17 cc) and most cells per slice area (79.18 cells/cm^2^). The control sorghum flour produced breads and cakes with low volume, poor crumb properties, and dense textures.	(91)
42	Bread	Incorporated amino acid and sugar pairs	The gluten-free bread and wheat bread were analyzed using gas chromatography and it was observed that the gluten-free bread lacks key flavor-generating compounds pyrazine and 2-acetyl-1-pyrroline. Out of incorporating different amino acid and sugar pairs the case of the proline and glucose pair was best for retaining flavor of bread.	(171)
43	Cakes	Used rice as alternate flour followed by incorporation of tapioca starch supplemented with resistant starch (a periodic and a functional dietary fiber)	Gluten-free cakes from rice flour and tapioca starch supplemented with resistant starch were investigated for physical and sensorial properties. With an increase in resistant starch level cakes, specific volume increased and was maximized at 15 g/100 g without affecting porosity values significantly. One cake batter was less elastic and thinner (viscosity decreased) with increased level of resistant starch concentration. Cake containing 20 g/100 g resistant starch was mostly preferred by panelists, although all cakes were acceptable.	(56)
44	Cookies	Substitution of corn flour with soy flour	Soy flour at different levels, 5, 10, and 15% was incorporated to produce gluten-free bread and it was found that bread with 15% soy flour had the best physical, chemical, and sensory qualities.	(172)
45	Muffins	Dietry fiber and Xanthan gum supplemented	Muffins prepared with 6% black carrot pomace dietary fiber concentrate and xanthan gum was reported to be most acceptable.	(173)
46	Bread	Corn, rice cream soup and tapioca starch	Effects of the combination of chestnut flour (40%) and sourdough (20%) fermentation was investigated on chemical, technological, and nutritional attributes of gluten-free breads. Breads prepared with chestnut flour and/or sourdough had lower volume with increased hardness compared to the control, but the combination of chestnut flour and sourdough contributed to reduce crumb grain heterogeneity. Reduction of staling was observed only at 5 days, even if a decrease in amylopectin fusion enthalpy was observed.	(174)
47	Cookies	Incorporation of Okara (by-product from soy milk production) along with commercial manioc flour	Different proportions of okara: 50; 30; 15; and 0% were used for developing gluten-free cookies and it was reported that incorporation of okara increased protein and fiber content. Overall acceptability increased significantly.	(175)
48	Turkish dessert Revani	Corn flour, rice flour, potato flour, corn starch, and tapioca starch	Celiac patients cannot consume revani because semolina contains gluten. Gluten-free revani was made using corn flour, rice flour, potato flour, corn starch, and tapioca starch and the recipes were developed with soy protein, pea protein and transglutaminase (TG) enzyme. A blend of rice flour and corn starch or a blend of corn flour, potato flour, and corn starch provided more regular distribution of air bubbles in revani. The sensory properties of gluten-free revani, made using a blend of corn flour and rice flour, were not affected by source of the protein and TG. It was observed that a combination of 62.5% corn flour and 37.5% rice flour with soy protein and TG was most suitable in making gluten-free revani.	(176)
49	Batter and Bread	Rice (50%), maize (30%) and quinoa flours with xanthan gum	Different levels of xanthan gum (1.5, 2.5, and 3.5%) and water (90, 100, and 110%) were added to a base formula of rice (50%), maize (30%), and quinoa flours (20%) during preparation of gluten-free batter and it was observed that more xanthan gum tended to produce batters of lower stickiness, adhesion, and cohesive-strength, yet of higher firmness, consistency, cohesiveness, and viscosity index. After baking, these loaves presented lower specific volume, lower crumb aw, pH, hardness, springiness, mean cell area, and void fraction; and higher (*p* < 0.001) chewiness, resilience, mean cell density, cell size uniformity, and mean cell compactness. It was reported that 110% WC and 1.5–2.5% xanthan gum yielded higher specific volume, lower crumb hardness, higher crumb springiness, and open grain visual texture in gluten-free loaves.	(177)
50	Bread	Quinoa flour, transglutaminase (TGase), and proteolytic enzymes	Scanning Electron Microscopy revealed that the quinoa starch granules (0.4–2 μm) in presence of TGase induced significant changes in dough and baked samples' microstructures. The overall acceptability of the breads was improved by TGase addition.	(178)
51	Cereal bar	quinoa, brown rice, flaxseed, dry fruits, and honey	The gluten-free cereal bars were developed for gluten-intolerant populations using dry raw materials (quinoa, brown rice, flaxseed, and dry fruits) and honey as a binding agent. Significant variation was observed physico-chemical and functional parameters of grains after dry heat treatment (80–100°C for 8–12 min) to grains (quinoa, brown rice, and flaxseed) prior to use in preparation of cereal bar. On the basis of sensory evaluation, a formulation with 50% honey level was found to be best.	(179)
52	Pasta	Blue/white maize flour, chickpea, and unripe plantain	The composite pasta exhibited acceptable cooking loss (9–11%); pasta with blue maize showed lower hardness and chewiness, but higher adhesiveness with dark color than its white maize-based counterpart. The addition of 75% blue maize flour yielded the highest total phenolic content retention after extrusion (80%) and cooking (70%) was good for health benefits.	(180)
53	Bread	Rice, tamarind gum	The batter properties and the quality of rice bread prepared using tamarind gum were equivalent or superior to those using other gums. The final viscosity after hydration of other gums and the pasting properties of rice flour with the tamarind gums were similar except for guar and xanthan gum. Cross-sections of rice bread showed that the addition of tamarind gum and pectin resulted in a fine appearance, but use of pectin was recommended due to its lower pH causing unpleasant sour taste and smell of the rice bread.	(181)
54	Fish Patties	Rice, corn, amaranth, or quinoa	Water and gluten-free flours (rice, corn, amaranth, or quinoa) were added to improve cooking yield, texture parameters and as an aid in improving quality attributes such as taste and juiciness. Cooking yields of patties containing gluten-free flours were higher than control and maximum values ranged between 91 and 93%. Patties made with amaranth or quinoa flour had higher hardness whereas cohesiveness and springiness were higher in corn and rice flour based patties. Response surface methodology optimized patties formulations.	(182)
55	chocolate cookies	rice flour and oligofructose-enriched inulin	Gluten-free chocolate cookies were characterized chemically, physically, and sensorially prepared with oligofructose-enriched inulin (25, 50, and 75%) as a partial substitute for rice flour. The chocolate cookie with 25% substitution of rice flour by inulin/oligofructose was as well accepted as the chocolate cookie with 100% rice flour (control) and the commercial cookie for most sensory properties.	(183)
56	Bread	Rice semolina, field beans with gums	The rice semolina supplemented with field bean semolina was used for formulation of gluten-free bread and for improving its final quality, choice of six factors: agar–agar, water, two types of gums (gum arabic and locust bean gum), and two types of starches (tapioca starch and corn starch) were incorporated. The results showed that specific volume of gluten-free breads increased significantly (*p* < 0.05) with the addition of gum arabic, tapioca and corn starches, and water. The gum arabic and water, as well as the interaction between them, had a significant effect on chewiness and springiness was affected by gum arabic, tapioca and corn starches, and water. Water and gum arabic had a significant (*p* < 0.0001) effect on all the properties of gluten-free breads. The final optimum formulation of rice/field bean contained 1.5% of gum arabic and 71.5% of water.	(184)
57	Biscuits	Foxtail millet and amaranth	Copra meal (15%) with germinated foxtail millet (85%) flour premixed and blended with amaranth in 60:40 proportions formulated gluten-free, calorie deficient composite biscuits. Successful calorie reduced in fat and binary (fat and sugar) amended biscuits had lower calorie (364.58 and 315.97 kcal/g, respectively) as compared with control (471.01 kcal/g).	(185)
58	Bread	Banana starch with maize starch and rice flour	The role of native and extruded banana starch, and a 1:1 native:extruded mixed banana starch composite (MB) was investigated in slowing down the starch digestibility of bread crumb and crust. It revealed a slowly digestible starch (SDS) increase from 1.09% (control) to 4.2, 6.6, and 7.76% in native, mixed, and extruded banana crumbs (fully gelatinized), respectively. A molecular size reduction resulted in highly gelatinized baked products rich in structurally driven SDS.	(76)
59	Bread	5–15% Egg white powder substituted gluten-free flour	Breads with egg white powder had larger specific volumes and more homogeneous texture than control gluten-free flours made breads. Adding egg white powder increased the elasticity of gluten-free batter and improved the texture properties of resultant bread during storage. The M200 egg white powder with more water-soluble protein aggregates yielded a more significant improvement in bread quality than general standardization sample P110.	(186)
60	Biscuits	Gluten-free flours, dough and biscuits	Addition of both fermented and unfermented *Agaricus bisporus* polysaccharide flour improved functional properties of composite gluten-free flours, dough, and biscuits, respectively, while addition of fermented *Agaricus bisporus* polysaccharide flour decreased viscosity property. Incorporation of both polysaccharide flours in composite gluten-free biscuit dough revealed a significant increase in rheological moduli (G′ and G′′) and a decrease in tan (δ). Supplementation of unfermented *Agaricus bisporus* polysaccharide flour increased thickness, whereas supplementation of fermented *Agaricus bisporus* polysaccharide flour increased diameter and spread ratio. All composite gluten-free biscuit formulations exhibited lower fracture strength and hardness compared to the control.	(187)
61	Cake	Potato starch, Corn starch, Broccoli leaf powder	New gluten-free mini sponge cakes fortified with broccoli leaves was developed. Broccoli leaf powder was a good source of nutritional components, including proteins and minerals, as well as bioactive compounds such as glucosinolates and phenolics. The antioxidant capacity of gluten-free mini sponge cakes significantly increased after incorporation of broccoli leaf powder. The addition of 2.5% broccoli leaf powder as a starch substitute resulted in an optimal improvement in the nutraceutical potential of gluten-free cakes without compromising their sensory quality.	(188)

## Conclusion

Worldwide wheat products are important staple foods. Wheat, along with related grains such as oats, barley, and rye, is the primary source of gluten in the diet. The gluten is essentially required for developing a strong protein network for providing the desired viscoelasticity of dough. About 1–2% of people (HLA-DQ2/8 genotypes) are diagnosed with celiac disease, an autoimmune condition triggering severe responses to the gluten proteins of wheat. The unique glutamine- and proline-rich sequences of gluten are involved in most wheat sensitivities. All safe gluten-free food must not exceed the level of gluten beyond 20 ppm. ELISAs are sensitive, specific, fast and suitable for the routine analysis of gluten, but LC-MS/MS of gluten marker peptides is the most promising alternative. A trend has been observed for gluten-free foods and beverages during the past two decades. Replacing gluten in gluten-free products requires utilizing a mix of recommended flours, proteins, hydrocolloids, and technologies in an attempt to replace gluten's multifunctional roles. The alternate raw materials have a sticky texture similar to gluten-rich wheat flours such as tapioca flour, corn meal, and potato starch are being used globally in order to meet the expectations of gluten-free products. The functionality of gluten-free dough has been improved through many treatments including acid/base, deamidation, cross-linking by oxidizing agents, and transglutaminase, proteolysis, disulphide bond reduction and high-pressure treatment. Enzyme treatments have improved gas holding and textural properties of gluten-free batters and breads. The teff flour was added to *Lactobacillus helveticus* for improving the perceived elasticity. The dietary fibers from flours, fruit and vegetable processing by-products, isolated ingredients, seeds, or mixtures have been used to improve nutritional quality and crumb porosity. It was noticed that the potential use of nutrient-dense raw materials, dietary fiber enrichment and technological processing decreased the glycemic response in gluten-free products. A proper optimization of physical processing such as germination, pressure, temperature improved gluten-free products. Molecular breeding approaches are one of the most promising options to downregulate coeliac-toxic proteins or mutate coeliac epitopes within individual proteins. The invention of biotechnological tools have made it feasible to produce gluten-free wheat by knocking down the gliadin gene using RNAi technology. There is a huge potential for gluten-free product marketing, keeping in view upcoming choices on product diversification and nutritional enrichment. More research is required for the production of gluten-free beverages/malts.

## Author Contributions

SR prepared the draft. AK and CC edited the manuscript.

### Conflict of Interest Statement

The authors declare that the research was conducted in the absence of any commercial or financial relationships that could be construed as a potential conflict of interest.
